# The Imprinted Retrotransposon-Like Gene *PEG11* (*RTL1*) Is Expressed as a Full-Length Protein in Skeletal Muscle from *Callipyge* Sheep

**DOI:** 10.1371/journal.pone.0008638

**Published:** 2010-01-08

**Authors:** Keren Byrne, Michelle L. Colgrave, Tony Vuocolo, Roger Pearson, Christopher A. Bidwell, Noelle E. Cockett, David J. Lynn, Jolena N. Fleming-Waddell, Ross L. Tellam

**Affiliations:** 1 CSIRO Livestock Industries, St Lucia, Queensland, Australia; 2 Department of Animal Sciences, Purdue University, West Lafayette, Indiana, United States of America; 3 Department of Animal, Dairy, and Veterinary Sciences, Utah State University, Logan, Utah, United States of America; 4 Department of Molecular Biology and Biochemistry, Simon Fraser University, Burnaby, British Columbia, Canada; Texas A&M University, United States of America

## Abstract

Members of the Ty3-Gypsy retrotransposon family are rare in mammalian genomes despite their abundance in invertebrates and some vertebrates. These elements contain a gag-pol-like structure characteristic of retroviruses but have lost their ability to retrotranspose into the mammalian genome and are thought to be inactive relics of ancient retrotransposition events. One of these retrotransposon-like elements, *PEG11* (also called *RTL1*) is located at the distal end of ovine chromosome 18 within an imprinted gene cluster that is highly conserved in placental mammals. The region contains several conserved imprinted genes including *BEGAIN*, *DLK1*, *DAT*, *GTL2 (MEG3)*, *PEG11 (RTL1)*, *PEG11as, MEG8, MIRG* and *DIO3*. An intergenic point mutation between *DLK1* and *GTL2* causes muscle hypertrophy in callipyge sheep and is associated with large changes in expression of the genes linked in *cis* between *DLK1* and *MEG8*. It has been suggested that over-expression of *DLK1* is the effector of the callipyge phenotype; however, *PEG11* gene expression is also strongly correlated with the emergence of the muscling phenotype as a function of genotype, muscle type and developmental stage. To date, there has been no direct evidence that *PEG11* encodes a protein, especially as its anti-sense transcript (*PEG11as*) contains six miRNA that cause cleavage of the *PEG11* transcript. Using immunological and mass spectrometry approaches we have directly identified the full-length PEG11 protein from postnatal nuclear preparations of callipyge skeletal muscle and conclude that its over-expression may be involved in inducing muscle hypertrophy. The developmental expression pattern of the *PEG11* gene is consistent with the callipyge mutation causing recapitulation of the normal fetal-like gene expression program during postnatal development. Analysis of the *PEG11* sequence indicates strong conservation of the regions encoding the antisense microRNA and in at least two cases these correspond with structural or functional domains of the protein suggesting co-evolution of the sense and antisense genes.

## Introduction

More than 45% of the mammalian genome is composed of repetitive elements, representing DNA transposons, long terminal repeat (LTR) retrotransposons, LINEs (Long Interspersed Nuclear Elements) and SINEs (Short Interspersed Nuclear Elements) [1]. Many of these are genetic relics of ancient transposition events and are generally thought to be inactive due to accumulated mutations and silencing by epigenetic genome ‘defense’ mechanisms, particularly DNA methylation [2]. However, in some instances these repetitive elements may influence the transcription of adjacent protein encoding genes [3]. DNA methylation is also involved in the regulation of genomic imprinting, causing genes to be mono-allelically expressed in a parent of origin specific manner; hence a relationship between the retroelements and the evolution of genomic imprinting mechanisms has been suggested [4], [5], [6], [7].

Ty3-Gypsy retrotransposons are rare in mammalian genomes despite their high abundance in some invertebrate and non-mammalian vertebrate classes [7]. A small family of nine mammalian genes with homology to the Ty3-Gypsy long terminal repeat retrotransposon Sushi-ichi from fugu has been identified [7], [8], [9]. These elements contain the gag-pol-like structure common to retroviruses, but have lost their long terminal repeats and presumably the ability to autonomously retrotranspose into the genome. Interestingly, two autosomal members of this gene family, paternally expressed gene 10 (*PEG10*) and paternally expressed gene 11 (*PEG11;* otherwise known as *RTL1*) are imprinted, being expressed only from the paternal chromosome, while five members map to the X chromosome [7]. The conservation of *PEG11* in placental mammals and its absence from syntenic chromosomal regions of nonplacental mammals suggests that during evolution the newly retrotransposed gene was co-opted for distinct functional roles within the placental mammals [7], [10].

*PEG11* is present in a conserved imprinted gene cluster spanning ∼1 Mbp which is located in sheep at the distal end of chromosome 18. The cluster contains at least nine imprinted genes including *BEGAIN*, *DLK1*, *DAT*, *MEG3 (GTL2)*, *PEG11 (RTL1)*, *PEG11as*, *MEG8, MIRG* and *DIO3* [5], [11], [12], [13], [14], [15]. This region controls the inheritance of the callipyge phenotype in sheep, which is characterised by postnatal muscular hypertrophy primarily localized to the hindquarters [12], [16], [17], [18]. The callipyge mutation is an A/G transition in a long range regulatory element located between *DLK1* and *GTL2* [11], [19], [20]. Unusually, the phenotype is only expressed by heterozygous lambs that inherit the mutation from their sire (i.e. the *NC^pat^* genotype). This non-Mendelian parent of origin inheritance pattern has been termed polar over-dominance [17], [21], [22].

The callipyge muscle hypertrophy phenotype is associated with enhanced expression of a core group of these imprinted genes linked in *cis* with the mutation. These include: (i) paternally expressed *DLK1 (DAT* is probably an alternatively polyadenylated transcript of *DLK1* [13]) and *PEG11*, and; (ii) the maternally expressed non-coding genes, *GTL2*, *PEG11as* and *MEG8* [11], [23], [24], [25], [26], [27], [28]. The paternal influence of the mutation on the muscle phenotype indicates that a paternally expressed gene is likely to be causal. Substantial evidence implicates *DLK1* as the effector of the phenotype and indeed *DLK1* is the only authenticated protein-encoding gene amongst these six genes [25], [28], [29], [30]. However, *PEG11* mRNA expression is also strongly up-regulated in affected muscles of paternal heterozygotes [23], [27], [31] suggesting that it also could be a contributor to the muscle hypertrophy in callipyge sheep.

Until now there has been little or no evidence that the *PEG11* retrotransposon-like transcript produces a protein, especially since the anti-sense transcript (*PEG11as*) contains six miRNA that have been shown to cause RISC-mediated cleavage of the *PEG11* sense transcript [31]. However, the paternally expressed ovine *PEG11* gene contains a conserved long open reading frame suggesting that it does produce a protein. The orthologous mouse *RTL1* protein has been localised in murine placenta by immunohistochemistry [32] although there were no details of the nature of the detected protein. We now report the direct identification of the full length ovine PEG11 protein isolated from callipyge skeletal muscle and demonstrate that its expression is substantially increased in the callipyge *NC^pat^* genotype.

## Results

### Organization of the imprinted genes located at the telomeric end of ovine chromosome 18

**Figure 1:**
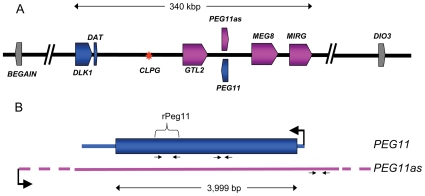
Diagrammatic representation of the organization of imprinted genes located at the telomeric end of ovine chromosome 18. (A) Representation of the approximate 1 Mbp region from *BEGAIN* to *DIO3*. The core imprinted genes affected by the callipyge mutation are colored while imprinted genes unaffected by the mutation are shown in grey. Paternally expressed genes are shaded blue and maternally expressed genes are shaded pink. The direction of transcription of each gene is indicated by the arrow in the gene symbol. Introns are not shown. The red asterisk denotes the position of the callipyge point mutation (*CLPG*). The precise lengths of the maternally expressed genes, which all produce non-coding RNAs, are unclear. The diagram is based on that deduced by [53] supplemented with annotation for a miRNA cluster (*MIRG*) deduced by comparative sequence analyses with the orthologous murine and human sequence regions. (B) Representations of the *PEG11* and *PEG11as* genes. A large black arrow denotes the direction of transcription of each gene. Small arrows show the relative positions of PCR primers. The region of *PEG11* expressed as a recombinant protein (rPEG11) is also shown. The precise length of the *PEG11as* gene is unclear but it extends beyond the *PEG11* gene in both directions (represented by broken lines).

Figure 1(a) shows a diagrammatic representation of the organization of imprinted genes located towards the telomeric end of ovine chromosome 18. The genes that showed altered expression as a result of the callipyge mutation are colored either blue or pink for paternally and maternally expressed transcripts, respectively. All core maternally expressed genes produce noncoding RNA. Only *DLK1* encodes an authenticated protein. The *PEG11* sequence is confined to a single exon with a 3,999 bp open reading frame potentially encoding a 151,028 Da protein. While there are some differences in the length of the *PEG11* gene in other species, notably rodents, all placental mammalian orthologs have maintained the single long open reading frame suggesting that a protein is produced. Figure 1(b) shows the relationship between *PEG11* and *PEG11as*. The 3′ and 5′ boundaries of the non-coding *PEG11as* transcript are undefined although it is known that the transcript includes the region corresponding to the *PEG11* transcript [11], [23], [31]. *PEG11as* also encodes six miRNA, mir-431, mir-433 (consisting of mir-433-5p and mir-433-3p i.e. both stem loops produce a functional miRNA), mir-127, mir-432 and mir-136 [31].

### Over-expression of PEG11 mRNA in callipyge semimembranosus and longissimus dorsi skeletal muscle during postnatal development

**Figure 2:**
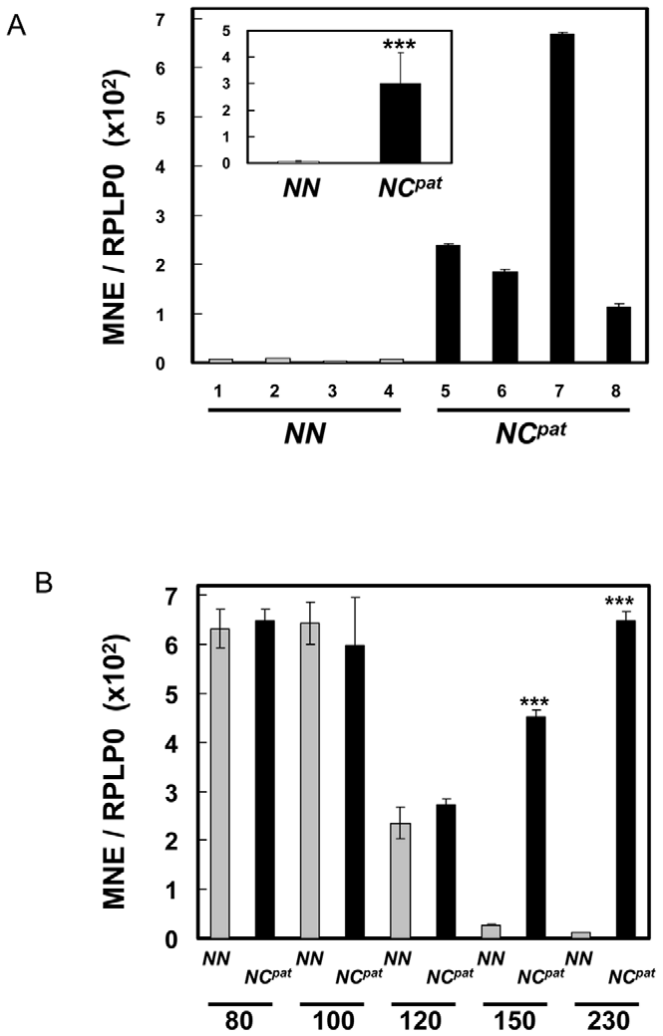
Relative expression levels of *PEG11*/*PEG11as* mRNA in wild type (*NN*) and callipyge (*NC^pat^*) genotypes during skeletal muscle developmental. Expression levels were measured by qRT-PCR and normalised to *RPLPO*. (A) *PEG11/PEG11as* mRNA expression in SM muscle taken at 12 weeks (230 days post-fertilisation) of age from four wild-type (*NN*) (grey) and four callipyge paternal heterozygote (*NC^pat^*) individuals (black). The mean expression values of the four individuals representing each genotype are illustrated in the inset. The qRT-PCR assay measured expression of both *PEG11* and *PEG11as* as the former is wholly contained within the latter. However, the contribution of *PEG11as* is relatively minor in these genotypes and thus the assay primarily measures expression of *PEG11* (see Materials and Methods). (B) *PEG11/PEG11as* expression in LD muscle during muscle development as a function of genotype. The samples were taken at 80, 100, 120, 150 and 230 days of development. Birth is at 147 days. The error bars denote the standard error of mean (n = 4). The asterisks denote significant (P<0.001) differences in expression levels between the *NC^pat^* (black) and *NN* (grey) genotypes at each developmental stage.

Gene expression analysis was used to measure the expression of *PEG11* in *semimembranosus* (SM) skeletal muscle from 12 week-old animals (i.e. 230 days post-conception; birth was at day 147) (Figure 2(a)). The SM muscle at this developmental time was known to undergo hypertrophy in callipyge (*NC^pat^*) compared with wild type (*NN*) sheep [12], [16], [17], [18]. Correspondingly, there was significantly enhanced expression (45 fold increase; P<0.001) of *PEG11* in the samples from *NC^pat^* sheep compared with wild type sheep. Some individuals of the same genotype were apparently more affected than others e.g. animal 7 in the *NC^pat^* group.

Developmental profiles for the expression of *PEG11* in *longissimus dorsi* (LD) skeletal muscle samples from fetal, newborn and adult sheep for the genotypes *NC^pat^* and *NN* are shown in Figure 2(b). The LD muscle also undergoes postnatal hypertrophy in callipyge (*NC^pat^*) compared with wild type (*NN*) sheep [12], [16], [17], [18]. In the *NN* genotype *PEG11* expression was relatively high in the fetal samples at 80 and 100 days of development, declined to a lower level at 120 days of fetal development and then declined much further in the two postnatal samples (150 and 230 d). In contrast, for the *NC^pat^* genotype *PEG11* expression declined just prior to birth (120 d) but then maintained a relatively high fetal-like expression level in the postnatal samples i.e. there were significantly higher levels of *PEG11* expressed in the two postnatal *NC^pat^* samples compared to *NN* samples at the same stages i.e. 150 d and 230 d. The enhanced expression of *PEG11* in the postnatal samples mirrored the emergence of the muscle hypertrophy phenotype in callipyge animals [33].

### Identification of PEG11 protein in callipyge semimembranosus skeletal muscle

**Figure 3:**
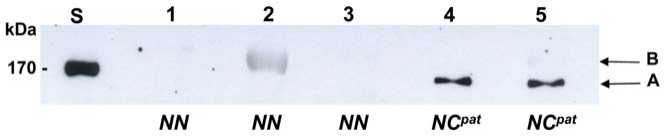
Immunoblot analysis for PEG11 protein in nuclear fractions from ovine SM skeletal muscle samples obtained from different individuals at 12 weeks of age. Nuclear extract (100 µg) from each muscle sample was separated by SDS-PAGE, transferred to nitrocellulose and probed with immunoaffinity-purified rabbit antibody raised to recombinant ovine PEG11 (rPEG11). Lanes 1–3, nuclear protein fractions from SM muscle obtained from three wild type (*NN*) individuals; lanes 4 and 5, two callipyge (*NC^pat^*) samples; lane S, protein size standards. Proteins designated A (∼Mr = 146,000 Da) and B (∼Mr = 190,000 Da) were identified by mass spectrometry analysis as PEG11 and myosin heavy chain, respectively.

For the identification of the PEG11 protein, samples of SM skeletal muscle were taken from callipyge and wild type sheep at 12 weeks of age (230 d of development) i.e. a stage when there was enhanced expression of *PEG11* mRNA and full expression of the hypertrophy phenotype in the *NC^pat^* genotype. Nuclear protein fractions were subjected to immunoblotting using an immunoaffinity-purified antibody raised in rabbits to recombinant ovine PEG11 (rPEG11) corresponding to a 162 amino acid region of the putative full length protein (see Fig. 1(b)). Figure 3 shows an immunoblot of SM skeletal muscle samples taken from three wild type (*NN*) and two callipyge (*NC^pat^*) animals. The band labelled A was only present in samples from the callipyge paternal heterozygote genotype (*NC^pat^*) but not wild type (*NN*) samples. This band corresponded to a protein of 146 kDa, which was consistent with the predicted size of the PEG11 protein deduced from its full length amino acid sequence (i.e. 151,028 Da; pI = 4.86) (ProtParam; http://us.expasy.org/tools). Regions corresponding to the immuno-reactive band were excised from SDS-PAGE gels, washed, and digested with trypsin. The resulting peptides were recovered and analysed by mass spectrometry. The weakly stained and diffuse 190 kDa band, designated B, was independently identified as myosin heavy chain, a prominent protein in skeletal muscle, using liquid chromatography electrospray ionization mass spectrometry/ mass spectrometry (LC-ESI-MS/MS) (ProtScore >7.5; statistical confidence >97%) (result not shown). The presence of this protein in one sample was probably an artefact of the protein fractionation procedure.

**Figure 4:**
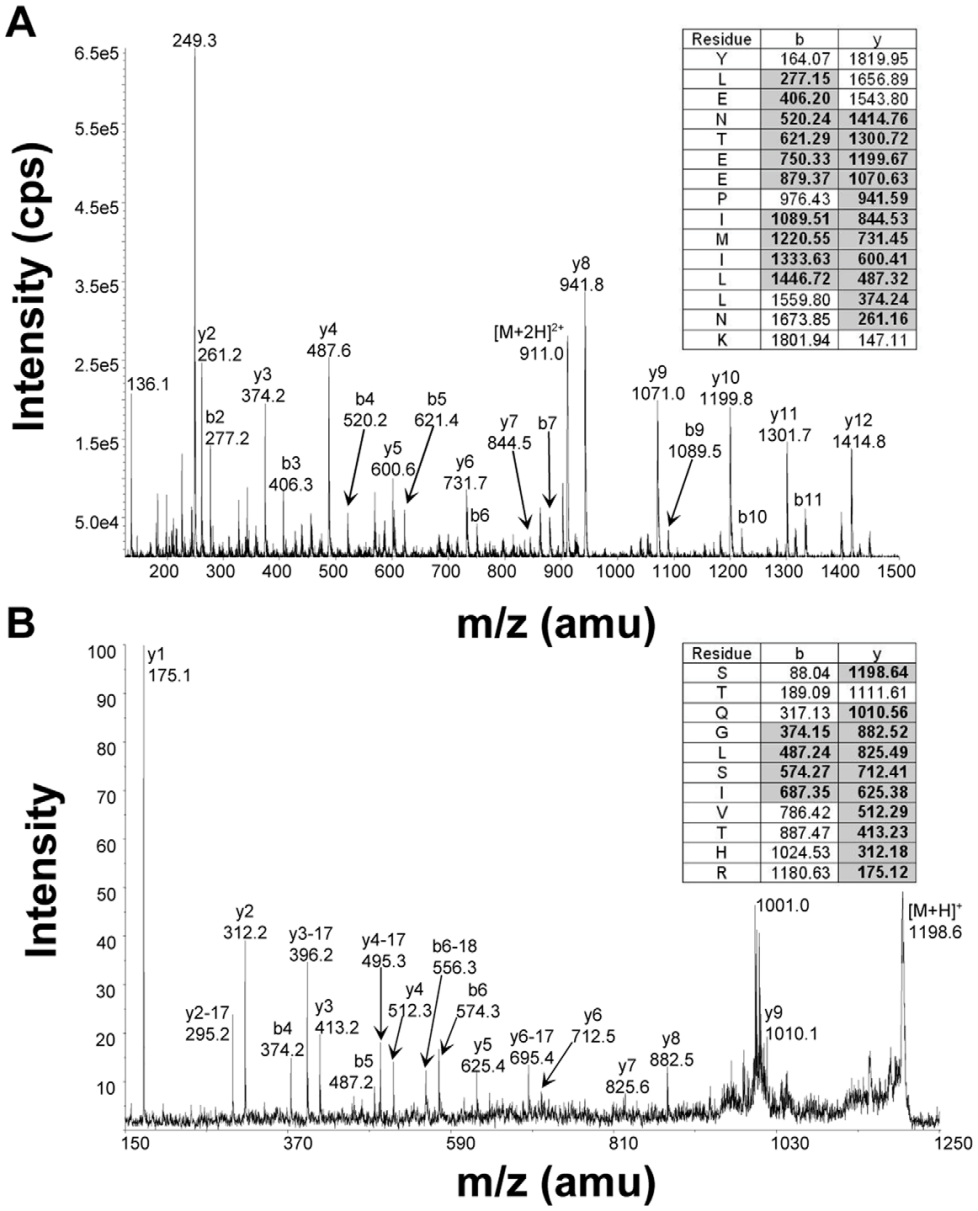
Mass spectrometry analyses of two peptides derived from the tryptic digest of protein contained within the PEG11 immuno-reactive region of the SDS-PAGE gel. Seven unique peptides were identified by using two parallel mass spectrometry approaches. Example product ion ms/ms spectra for peptides identified by each technique are shown. (A) LC-MALDI-MS/MS, and; (B) LC-ES-MS/MS. For each peptide, the amino acid sequence is tabulated along with the theoretical m/z values corresponding to the *y* and *b* ions. The experimentally detected sequence ions are denoted by bold typeface and grey shading (insets). A selection of the sequence ions is labelled in the ms/ms spectra.

**Figure 5:**
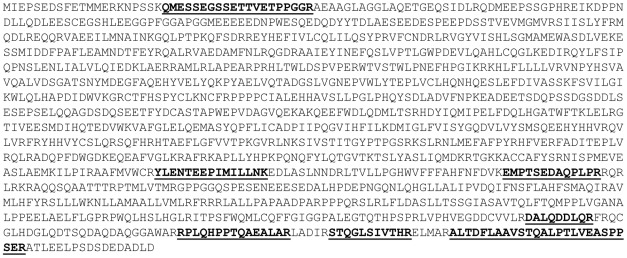
Sequence coverage of PEG11 peptides. The peptides sequenced by LC-MALDI-MS/MS and ESI-LC-MS/MS are bolded and underlined. The combined peptide sequences represented 8.2% of the ovine PEG11 sequence. The putative N-terminus was deduced from the longest open reading frame of the mRNA sequence in conjunction with the marked DNA sequence conservation transition occurring 5′ to the putative initiating methionine.

Two parallel approaches were undertaken to determine peptide amino acid sequences from band A. First, liquid chromatography matrix assisted laser desorption ionization time-of-flight/ time-of-flight mass spectrometry (LC-MALDI-TOF-TOF) analysis of the tryptic digest was performed with manual searching and identification of theoretical PEG11 tryptic peptides. Second, a targeted analysis using hypothetical multiple reaction monitoring (MRM) and an MRM-initiated detection and sequencing workflow on a linear ion trap mass spectrometer was employed (LC-ESI-MS/MS). Database searching using ProteinPilot™ (Applied Biosystems) software was conducted in which a customised FASTA file was created to include the putative PEG11 protein sequence. The LC-MALDI-TOF MS/MS analysis detected and sequenced five peptides while the LC-ESI-MS/MS analysis detected and sequenced four peptides (Table 1). Two peptides were identified by both methodologies. All peptides detected using these dual approaches were derived from ovine PEG11. Figure 4 shows one example spectrum of each approach. The combined peptide sequences corresponded to 8.2% of the predicted PEG11 amino acid sequence (Fig. 5). This coverage provided unequivocal identification of the protein. The absence of the full complement of peptides was probably due to several factors including the inability to detect some individual peptides by mass spectrometry due to peptide amino acid composition bias, extremes of peptide length, and potential post-translational modifications. All peptides were preceded by and ended in K or R, as expected from the specificity of trypsin. The identification of proximal N-terminal and C-terminal peptides indicated that the PEG11 protein was likely to be full length or near full length in sequence, as predicted from its size upon immunoblotting. Attempts at Edman sequencing using a Procise Protein Sequencer to identify the N-terminus were unsuccessful suggesting that it was chemically modified. The N-terminus was assigned to the sequence commencing MIEP as a consequence of predicted mRNA sequence conservation in regions 3′ to this putative ATG start site and poor conservation in the region 5′ to this site.

**Table 1:** Ovine PEG11 peptides detected by mass spectrometry^1^.

Detection method	Peptides	Theor. MW (Da)	Exp. MW (Da)	Δm (Da)
**LC-MALDI-MS/MS**	DALQDDLQR	1072.51	1072.48	0.03
	STQGLSIVTHR	1197.65	1197.62	0.03
	RPLQHPPTQAEALAR	1683.92	1683.91	0.01
	QMESSEGSSETTVETPPGGR	2064.89	2064.89	0.00
	ALTDFLAAVSTQALPTLVEASPPSER	2683.41	2683.33	0.08
**ESI-LC-MS/MS**	EMPTSEDAQPLPR	1469.68	1469.03	0.65
	STQGLSIVTHR	1197.65	1197.60	0.05
	YLENTEEPIMILLNK	1818.94	1819.30	0.36
	ALTDFLAAVSTQALPTLVEASPPSER	2683.41	2682.91	0.50

1 Data from the seven different peptide fragments identified by LC-MALDI-MS/MS and LC-ESI-MS/MS are tabulated with their corresponding theoretical and experimental peptide masses (Da), as well as the difference in mass (Δm).

### Impact of antisense miRNA on the coding potential of PEG11

**Figure 6:**
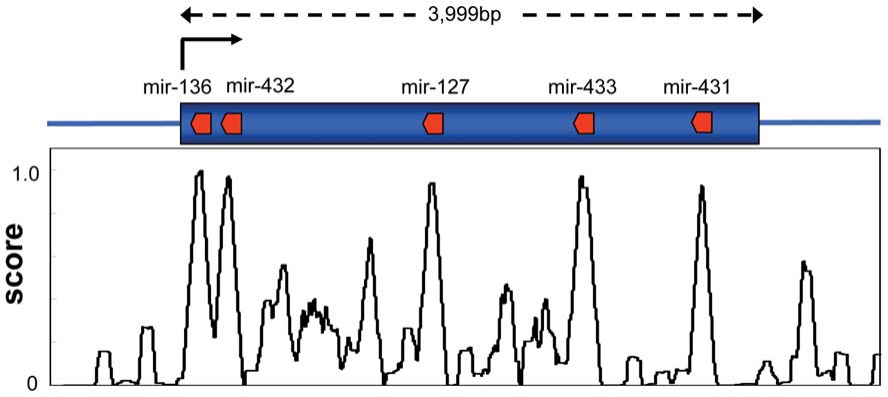
Conserved regions of the ovine *PEG11* gene correspond with antisense miRNA. The *PEG11* open reading frame is shown in blue while the locations of antisense miRNA (mir-431, mir-433, mir-127, mir-432 and mir-136) are shown in red. The black arrow denotes the direction of transcription of *PEG11*. The graph shows the extent of *PEG11* gene conservation using the UCSC PhastCons Conserved Elements 17-way Vertebrate Multiz Alignment and Conservation tool [34].

Figure 6 shows regions of localized conservation in the available mammalian *PEG11* nucleotide sequences (except rat and mouse), which were measured using the UCSC PhastCons Conserved Elements Vertebrate Multiz Alignment and Conservation tool [34]. Also shown are the relative positions of six conserved miRNA present in the *PEG11as* gene i.e. mir-431, mir-433 (consisting of mir-433-5p and mir-433-3p i.e. both stem loops produce a functional miRNA), mir-127, mir-432 and mir-136. The mouse and rat sequences were not included as they additionally each contain two large non-homologous insertions which make alignments problematic. The regions of high conservation in the *PEG11* gene corresponded precisely with the antisense miRNA. Thus, there were strong constraints imposed on the PEG11 protein sequence caused by regions encoding conserved miRNA antisense to the *PEG11* gene. Examination of the rates of nonsynonymous (d_N_) and synonymous (d_S_) substitutions using all available mammalian full length *PEG11* sequences (except rat and mouse) revealed that d_N_/d_S_ values were significantly less than one thereby demonstrating that purifying selection was operating across the whole of the gene [10] (and our unpublished results). A similar but more focused examination of each *PEG11* region corresponding with each miRNA was confounded by the assumption in these analyses that synonymous substitutions in these regions represent neutral evolution. This was clearly not the case due to the conservation of miRNA sequence in the antisense strand. To overcome this limitation an average value of d_S_ over the whole of the gene was used for analysis of each region corresponding to the antisense miRNA. The ratio of d_N_ (specific to the *PEG11* region defined by each antisense miRNA) to d_S_ (calculated across the whole of the gene) was calculated for all six ovine miRNA regions. In almost all cases, d_N_/d_S_ values were considerably less than the value calculated for the whole gene indicating that there was relatively greater purifying selection in the regions containing antisense miRNA compared with the whole gene (result not shown). The exception was the region corresponding to mir-431 where the value of d_N_/d_S_ was slightly greater than one, likely indicating relaxed selective constraint.

**Figure 7:**
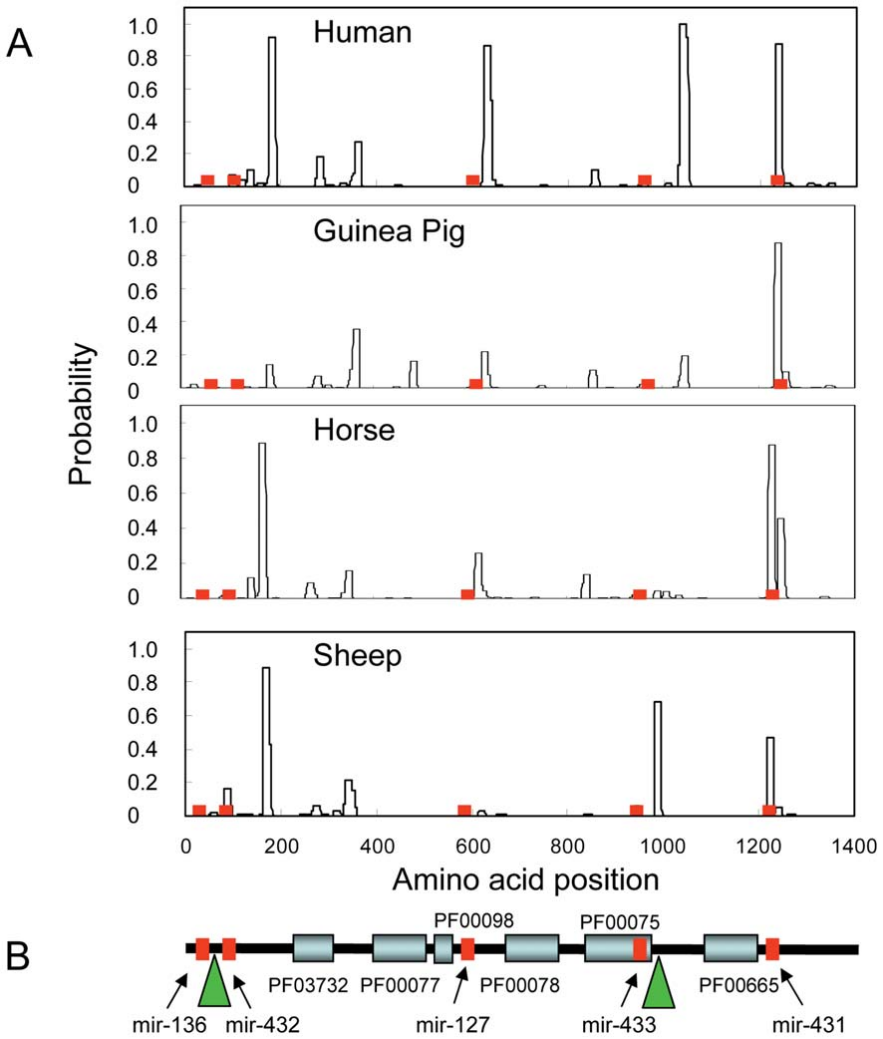
Structural domains in the PEG11 protein. (A) Probability of coiled coil formation. All sequences were extracted from the UCSC genome browser (http://genome.ucsc.edu/). Coiled coils were predicted using *Coils* and a 14 amino acid window [52]. The relative positions in the PEG11 protein corresponding with regions encoding antisense miRNA are shown in red. (B) Structural domains common to mammalian PEG11 proteins. The diagram is based on the ovine sequence but is representative of mammalian sequences. Structural domains were identified by searching the Pfam protein families database [51]. PF03732 (capsid-like or retrotransposon gag protein domain; Expect score = 3.4e−11); PF00077 (retroviral aspartyl protease domain; Expect score = 0.33); PF00098 (zinc finger knuckle; Expect score not significant); PF00078 (reverse transcriptase domain; Expect score  = 1.4e−5); PF00075 (RNaseH; Expect score not significant) domain; PF00665 (integrase core domain; Expect score not significant). Mutations to key residues in some of the PEG11 retrotransposon domains have compromised the significance of identification of some Pfam families, which are often biased in their weightings of catalytic residues. The positions corresponding to regions encoding antisense miRNA are shown in red. The green triangles denote positions where there are large insertions of repetitive sequence in the murine and rat protein sequences. The sequence insertions at each site for these two species are not conserved in sequence or length.

Figure 7(a) shows the probabilities of coiled coil formation in the human, guinea pig, horse and sheep PEG11 protein sequences. The regions of high probability for coiled coil formation highlight localised differences between the species. The peaks were generally in the same relative locations except that species specific combinations of the peaks occur. Whereas human had four major peaks, guinea pig had one while the artiodactyl species, horse and sheep, had two or three, respectively. The only conserved peak in all four species corresponded exactly with a region encompassing the conserved antisense miRNA, mir-431. In this instance the presence of the conserved antisense miRNA in the *PEG11* gene placed constraints on the PEG11 protein sequence in this region possibly implicating the conserved coiled coil structure in the function of PEG11. Inclusion of the corresponding murine and rat profiles was problematical as each of these rodent sequences had insertions of two large repetitive regions (green triangles in Figure 7(b)) and the sequences and lengths of these regions were quite different in these two species.

Figure 7(b) shows a diagrammatic representation of the domain structure of the ovine PEG11 protein based on conserved structures present in all mammalian sequences. Six structural domains typical of a retrotransposon were identified i.e. capsid-like or retrotransposon gag protein domain (PF03732), retroviral aspartyl protease domain (PF00077), a reverse transcriptase domain (PF00078), an RNaseH domain (PF00075) and an integrase core domain (PF00665), although only PF03732 and PF00098 were significant (Expect scores = 6.6e−11 and 1.4e−5, respectively; http://pfam.sanger.ac.uk). There was also a zinc finger knuckle (PF00098) closely associated with the retroviral aspartyl protease domain. As has been previously reported, mutations in many of the catalytic sites of the reverse transcriptase, RNaseH and integrase domains probably inactivated these activities [10]. These mutations were largely responsible for the lack of significant Expect scores associated with the pfam protein domain analysis. However, the capsid-like or retrotransposon gag protein domain and retroviral aspartyl protease domain largely maintained essential residues suggesting that they may be functional [10] (our unpublished results). The immunoblot and peptide mass spectrometry data, which demonstrated that the PEG11 protein was full length, suggested that the aspartyl protease domain was either inactive or did not have autolytic activity. Notably, there was only one instance where a coding region corresponding to an antisense miRNA was present within one of these domains i.e. the RNaseH domain (PF00075). Furthermore, none of the coiled coil regions was present within any of these functional domains. The relative positions of the large murine and rat repetitive insertions are also shown and these also do not correspond with any of the functional domains.

## Discussion

The conservation of a long open reading frame in the *PEG11* gene and evidence for purifying selection suggested that it encodes a protein. The current study has demonstrated that *PEG11* is transcribed and produces a full length 151,028 Da protein in callipyge skeletal muscle. This is the first definitive demonstration that this mammalian retrotransposon related gene produces a corresponding protein. There has been one report, using a mouse genetic model of RTL1 over-expression, showing perinuclear immunolocalisation of RTL1 in capillary endothelial cells of the placenta [32]. Gene targeting of *RTL1* combined with paternal or maternal inheritance of the disrupted allele resulted in loss or over-expression of *RTL1* expression, respectively [32]. Both circumstances generated placental abnormalities contributing to late-fetal and /or neonatal lethality phenotypes, respectively.

The expression of *PEG11* mRNA was markedly increased (45 fold) in callipyge (*NC^pat^*) SM muscle, which correspondingly shows extreme muscle hypertrophy two to three months after birth [16], [33], [35]. The reason why some animals were more affected than others is not clear but could hint at the existence of modifier genes or variation in the extent of allele specific imprinting. The over-expression of *PEG11* is associated with the emergence of the callipyge skeletal muscle hypertrophy phenotype as a function of genotype, developmental stage and muscle type thereby suggesting a possible causal role. It has been reported that *PEG11* mRNA expression is also up-regulated in *supraspinatus* callipyge skeletal muscle [23], [36], a muscle showing no hypertrophy [35], and therefore it was suggested that PEG11 is not involved in the induction of muscle hypertrophy in callipyge animals. However, the up-regulation is relatively small and the absolute values are much less than in affected muscles. Thus, this observation does not preclude a potential causal role of PEG11 in the induction of hypertrophy in callipyge skeletal muscle. Indeed, subtle phenotypic effects of the mutation in SS muscle are apparent [37].

There are unusual genetic mechanisms associated with the inheritance of the callipyge phenotype as the muscle hypertrophy phenotype is only present in paternal heterozygote animals and first becomes apparent approximately one month after birth primarily in muscles located toward the rear of the animal [12], [16], [18], [22], [33]. An elegant genetic model has been proposed to explain this observation [11]. The model suggests that the balance between a paternally expressed effector and a maternally expressed *trans*-acting repressor dictates the phenotypic outcome. In the paternal heterozygote the effector is influenced by the mutation acting in *cis* causing its marked over-expression relative to the maternally expressed repressor. The excess of effector in this genotype thereby induces the muscle hypertrophy. Conversely, in the *C^mat^N* genotype the mutation acts in *cis* to increase expression of the maternally expressed repressor but it has no influence on the effector which it is not expressed from the wild type paternal allele. Hence there is no phenotype. In the homozygote (*C^mat^C^pat^*) both the paternally expressed effector and maternally expressed repressor are up-regulated but there is no imbalance between effector and repressor, and therefore no phenotype.

The only two paternally expressed genes in the core of the imprinted locus in which the mutation lies, and hence potential effectors of the phenotype, are *DLK1* and *PEG11*. There is significant evidence suggesting that DLK1 is the effector. First, the increased expression of *DLK1* as a function of genotype, development, muscle type and muscle fibre type is also strongly associated with the expression of the hypertrophy phenotype [11], [26], [28], [36]. Second, DLK1 has been implicated in the control of cell proliferation and differentiation [38]. Third, transgenic mice over-expressing *DLK1* using a myosin light chain 3F promoter were characterised by mild skeletal muscle hypertrophy [29]. However, there is also evidence that is inconsistent with DLK1 being the effector. First, a murine *DLK1* knockout resulted in obesity but no reported muscling phenotype [39]. Second, constitutive expression of *DLK1* in the murine myogenic cell line C2C12, which does not express endogenous *DLK1*, did not affect the differentiation of these cells into multinucleated myotubes [40]. Third, over-expression of *DLK1* (using its endogenous regulatory elements) in transgenic mice resulted in growth enhancement but the mice failed to survive early life and showed no signs of muscle hypertrophy [41]. Thus, the cumulative evidence makes it difficult to differentiate between DLK1 and PEG11 as the potential effector of the callipyge skeletal muscle hypertrophy. Indeed, it is possible, and perhaps even likely, that both proteins may contribute to the callipyge phenotype. This hypothesis could be tested using transgenic mice over-expressing both genes in the same skeletal muscle tissues or by using a muscle cell line transfected with over-expression constructs for both genes.

The nature of the maternally expressed repressor is unclear although it is noted that only non-coding RNA are maternally expressed from the core of this imprinted locus [11], [27]. These maternally expressed non-coding genes are strongly up-regulated in the *C^mat^N* and *C^mat^C^pat^* genotypes, both of which do not show a muscle hypertrophy phenotype. Thus, the presence of the callipyge mutation on the maternal allele results in *cis-*mediated enhanced expression of these noncoding genes, which could act as *trans*-acting repressors of the effector produced by either the *C^mat^N* or *C^mat^C^pat^* genotypes, thereby neutralising the influence of the effector. The miRNA embedded in the maternally expressed *PEG11as* gene cause RISC-mediated cleavage of the *PEG11* transcript [31] and hence are ideally placed to be the putative maternally expressed *trans*-acting repressor of the paternally expressed *PEG11* effector. It is therefore possible that the mutation on the paternal allele of the *NC^pat^* genotype acting in *cis* causes up-regulation of *PEG11* expression whilst maintaining only a low wild type expression level of *PEG11as* from the maternal chromosome. This would result in a markedly increased ratio of *PEG11* to *PEG11as* in the paternal heterozygote and therefore a predominant influence of PEG11 as the effector of the callipyge phenotype. In the homozygote, enhanced expression of both *PEG11* and *PEG11as* would occur through the *cis*-mediated action of the mutation; however their ratio may not significantly alter thereby resulting in little change in the level of *PEG11* that is not potentially bound to miRNA derived from *PEG11as*. This model could explain the absence of phenotype in the *C^mat^C^pat^* and *C^mat^N* genotypes and the presence of the phenotype in the paternal heterozygote.

An alternative mechanism to explain the unusual callipyge genetics could be that the ∼50 miRNA associated with the maternally expressed gene, *MIRG* [42], act in an analogous fashion to the miRNA embedded in *PEG11as* except that they target the *DLK1* transcript. In this case it is hypothesised that DLK1 is the effector of the phenotype. Indeed, bioinformatics predictions indicate that some of the *MIRG* miRNA may target *DLK1* [13]. DLK1 is an atypical Notch-like ligand that is expressed on the surface of cells or present in a soluble circulating form. One possibility is that the miRNA derived from *MIRG* preferentially target *DLK1* splicing variants and thereby change the ratio of the constitutively membrane-bound DLK1 C2 variant to variants that can produce soluble circulating forms of DLK1, which act in a paracrine like manner. This possibility could result in a miRNA mediated molecular switch that changes the nature of signalling mechanisms mediated by DLK1. Definition of the targeting specificities of the multiple miRNA expressed from the *MIRG* gene as well as the nature of DLK1 cellular signalling are required to substantiate this hypothesis.

The *PEG11* gene is only present in placental mammals and it is suggested that its insertion in an eutherian ancestor was responsible for the development of imprinting at this locus probably as a by-product of a genome defence mechanism protecting against retrotransposon insertions [5]. The conservation of the *PEG11* open reading frame despite the acquisition of mutations that inactivate most of the retrotransposon activities suggests that this protein was co-opted for mammalian function. A murine *RTL1* (*PEG11)* knockout upon paternal transmission in hemizygous animals results in fetal death which is accompanied by placental defects specifically involving loss of fetal capillaries at the fetal-maternal interface [32]. Conversely, over-expression of *RTL1* as a result of deficiency of *RTL1as* upon maternal transmission in *RTL1* knockout mice resulted in placentomegaly and neonatal lethality. Therefore, it was concluded that during mammalian evolution RTL1 had acquired unique roles in placentation that were exquisitely sensitive to RTL1 dose. Genomic imprinting may have been acquired at this locus to regulate the dose of RTL1 during embryonic development. Remarkably, in callipyge sheep the gene is strongly up-regulated in the *NC^pat^* genotype but only in select muscles during postnatal development. This up-regulation, unlike that experimentally induced during murine embryonic and fetal development, does not compromise animal viability, and thus dosage effects must not be critical during postnatal development.

The function of PEG11 in normal fetal skeletal muscle is unclear as is its precise role, if any, in generating muscle hypertrophy when it is over-expressed in callipyge skeletal muscle. One possibility is that the retroviral GAG-like domain may promote cell fusion leading to enhancement of the formation of multinucleated myotubes and thereby promoting muscle hypertrophy. However, the location of PEG11 in the nucleus mitigates against this role. Another possibility, which is an extension of its placental role [32], is that PEG11 may be responsible for increasing capillary growth in skeletal muscle. This would be consistent with the presumed increased vascular demands of hypertrophied muscle. The normal postnatal down-regulation of *PEG11* in skeletal muscle is also consistent with a primary role in late fetal development. The callipyge mutation appears to cause recapitulation of a fetal-like *PEG11* expression profile postnatally, suggesting that the muscle hypertrophy phenotype is associated with the inappropriate postnatal extension of the PEG11 function.

The conserved long open reading frame in the *PEG11* gene indicates that there is an evolutionary requirement to maintain the encoded functional protein domains even though mutations to key residues in some of these domains may have inactivated their catalytic functions. What may be retained is the ability to interact with other proteins or nucleic acids. The retroviral aspartyl protease domain has maintained essential catalytic residues [10]; however, the presence of the full-length form of PEG11 in callipyge skeletal muscle suggests that it has little autolytic activity. One of the functions of this protease activity in authentic retrotransposons is to process the retrotransposon polyprotein into its various functional domains. Further experiments are required to examine the potential proteolytic activity and identify protein binding partners of PEG11 to establish its functional roles.

The highly conserved miRNA present in *PEG11as* place strong coding sequence constraints on the *PEG11* protein encoding gene. The functional implications of this effect on the PEG11 protein are unclear as the antisense miRNA generally do not correspond with the known retrotransposon-like domains or other structural features. However, there are two exceptions. First, the region of PEG11 corresponding with that encoding antisense mir-431 is associated with propensity for coiled coil formation. Whilst coiled coils are a general feature of the PEG11 protein sequence, the region corresponding to that encoded by antisense mir-431 is highly conserved in its propensity for coiled coil formation suggesting an important function possibly involving protein-protein interactions. Interestingly, most other regions in the PEG11 sequence that corresponded with antisense miRNA showed indications of purifying selection relative to the remainder of the protein. This indicates that mutations in these regions are deleterious to evolutionary fitness. However, the antisense mir-431 region in *PEG11* showed relaxation of selective constraint perhaps indicating that coiled coil formation in this region can better tolerate amino acid substitutions and is of greater biological importance than the constraining influence of antisense mir-431 in the coding sequence of *PEG11*. Second, the mir-433 antisense sequence corresponded to part of the RNaseH domain (PF00075) but mutations to its active site residues have probably inactivated its catalytic function. Thus, the collective impacts of the antisense miRNA on PEG11 protein activities are unclear. However, it is likely that there has been co-evolution of *PEG11* and *PEG11as* genes to accommodate the presence of mir-431 and mir-433 in the structural and functional features of the PEG11 protein.

The primary role of the miRNA is certainly to directly regulate *PEG11* mRNA by RISC-mediated cleavage of the *PEG11* sense transcript [31]. Thus, a trans-mediated effect of the maternally expressed miRNA can have considerable impact on a target gene that is paternally expressed. Presumably this unusual regulatory architecture is required to regulate gene dosage in a situation where there is need for an optimal dose, but too much or too little result in adverse biological outcomes.

In conclusion, an authentic full length PEG11 protein is over-expressed in callipyge skeletal muscle and this retrotransposon-like protein may be involved in generating the muscle hypertrophy characteristic of callipyge sheep.

## Materials and Methods

### Ethics statement

All animals were reared and euthanased in a humane manner in accordance with the approved Utah State University Animal Care and Use Committee protocols.

### Biological samples

Skeletal muscle samples were obtained from Dorset/Suffolk/Rambouillet cross-bred sheep raised at Utah State University. Matings were conducted to produce offspring comprising wild type (*NN*) and callipyge (*NC^pat^*) genotypes. The genotypes of all animals were confirmed by restriction fragment length polymorphism analysis [19], [20]. Samples were taken of *semimembranosus* (SM) skeletal muscle from new born lambs 2–3 days after birth (150 d) and lambs at 12 weeks of age (230 d). Samples of *longissimus dorsi* (LD) skeletal muscles were also taken from fetal lambs at 80, 100 and 120 days of gestation, new born lambs 2–3 days after birth (150 d) and lambs at 12 weeks of age (230 d). Birth is at day 147. The effects of genotype on the characteristics of these muscles and gene expression within the muscle have been previously reported [11], [16], [17], [18], [19], [20]. All muscles were dissected from the animal within 15 minutes of euthanasia, weighed and samples collected at equivalent pre-determined sites before freezing in liquid nitrogen.

### RNA isolation and cDNA synthesis

Total RNA was extracted from 4 g of tissue following pulverisation under liquid nitrogen in Trizol reagent (Invitrogen). A 100 µg aliquot of each RNA sample was treated with DNase 1 (Ambion) and further purified using an RNeasy Midi Kit (Qiagen) including a second DNAse 1 on–column treatment to remove any residual traces of genomic DNA. The RNA was quantified by spectrophotometric measurements at 260 nm and 280 nm and its purity verified by the OD_260_/OD_280_ ratio (>1.8) and integrity validated by visualisation on an agarose gel. cDNA synthesis was undertaken with 5 µg of the purified RNA using MMLV Superscript III reverse transcriptase (Invitrogen) and an anchored oligo-T_18_ primer combined with random hexamers [43]. A number of controls validated the absence of significant quantities of residual genomic DNA. First, DNAse 1 treated total RNA was subjected to PCR using *PEG11* primers and no significant expression was detected (Ct>35). Second, PCR products were generated from the cDNA using primer pairs specific for nine unrelated genes. These primers were selected to produce differentially sized amplicons for genomic DNA and cDNA. In all instances only amplicons characteristic of cDNAs were amplified.

### Quantitative real time RT-PCR (qRT-PCR)

qRT-PCR measurements were performed using the SYBR green system in an ABI prism 7900 Sequence Detection System (PE Applied Biosystems, Foster City, CA). Primer pairs were designed with DS Gene software (ver 1.5) (Accelrys)) using publicly available ovine and bovine sequence information (NCBI, http://www.ncbi.nlm.nih.gov/) and the Interactive Bovine In Silico Single Nucleotide Polymorphism (IBISS, http://www.livestockgenomics.csiro.au/ibiss/) systems (Table 2). A constant amount of cDNA derived from 10 ng of total RNA was used for each qRT-PCR measurement and four technical replicates were performed for each gene. Each qRT-PCR (5 µl total volume) contained: 2.5 µl of 2x SYBR Green Master Mix (Applied Biosystems); 0.25 µl of each primer giving a final concentration of 450 nM each; 1.0 µl water, and; 1.0 µl of a 1/10 dilution of the cDNA template. Cycling conditions were 40 cycles of 95°C for 15 s and 60°C for 1 min. At the completion of each run, a dissociation melt curve analysis was performed to ensure the presence of a single specific amplicon. Each qRT-PCR assay was validated by amplicon size and sequence. Acidic ribosomal protein P0 (*RPLPO*) was used as the reference gene [27], [28] following demonstration that its expression was constant in all samples for the same input cDNA. Data analyses were performed using Q-gene qRT-PCR analysis software (Gene Quantification (http://www.gene-quantification.info/) [44] and results were expressed as Mean Normalized Expression (MNE) relative to the reference gene. The efficiency (E) of amplification for each assay was determined (*RPLP0*, E = 1.96; *PEG11/ PEG11as,* E = 1.90) and applied to calculation of MNE for each sample. Gene symbols used in the context of a gene or mRNA are shown in italics while normal text denotes use in the context of the corresponding protein.

**Table 2:** Oligonucleotide sequences.

Gene (accession number)	Forward primer (position)	Reverse primer (position)	Amplicon size (bp)
*PEG11/PEG11as* (AF354168)	5′ cttccactctccctactgcct 3′ (151950–151930)	5′ gcatccacaggttcccac 3′ (151657–151674)	294
*PEG11as* (AF354168)	5′ tcggggctgaggtgggaatctc 3′ (154009–154030)	5′ cccagctgaagggatcacagcc 3′ (154108–154087)	100
*rPEG11* (AF354168)^1^	5′ *atggatcc*gctttctactcccgcaacatct 3′ (150812–150791)	5′ *aggaattcctcagtgatggtgatggtgatg*gaatatctggtccacgggtatc 3′ (150327–150348)	524
Acidic ribosomal protein (AF013214)	5′ caaccctgaagtgcttgacat 3′ (550–570)	5′ aggcagatggatcagcca 3′ (776–759)	226

1 Flanking sequences for a hexa-His tag and the engineered restrictions sites for *Bam*H1 and *Eco*R1 are shown in italics.

The measurement of the expression of *PEG11* mRNA using qRT-PCR can be complicated by the simultaneous expression of the antisense transcript, *PEG11as,* which fully encompasses the region corresponding to the *PEG11* gene [11], [23], [27]. Due to this genetic architecture, it was not possible to specifically detect only *PEG11* expression; rather the qRT-PCR assay detected both *PEG11* and *PEG11as*. An assay specific for *PEG11as* was developed in a region not present within the *PEG11* transcript. Using this independent assay it was demonstrated that the up-regulation of *PEG11* greatly predominated over *PEG11as* expression in affected skeletal muscle from *NC^pat^* sheep [27]. Indeed, when expression of *PEG11as* was specifically measured in *NC^pat^* SM muscle at birth (150 d of development), it was expressed 18.42±6.33 fold less than for the assay that measured both *PEG11* and *PEG11as.* This result indicated that increased expression of *PEG11* was strongly predominant over *PEG11as* in the *NC^pat^* genotype. Thus, the qRT-PCR assay for *PEG11* is a good approximation for *PEG11* expression in callipyge samples.

### Production of recombinant *PEG11*

Ovine *PEG11* sequence (GenBank accession AF354168) was employed to design PCR primers for amplification of a region encoding a 162 amino acid fragment (Fig. 1; Table 2). The reverse primer also contained sequence encoding a C-terminal Hexa-His affinity tag. The 524 bp amplicon was cloned into the glutathione S transferase gene fusion vector pGEX2T (GE Healthcare Bio-Sciences), via the shuttle vector pGEM-T (Promega). Nucleotide sequences were confirmed by DNA sequencing (Australian Genome Research Facility, Brisbane). Ovine recombinant PEG11 (rPEG11) was expressed from the pGEX2T vector according to the manufacturer's instructions (GE Healthcare Bio-Sciences). The rPEG11, which was initially present in inclusion bodies, was solubilised in 8 M urea, 100 mM NaH_2_PO_4_, 10 mM Tris-HCl pH 8.0, and 5 mM β-mercaptoethanol and purified by nickel-nitrilotriacetic acid (Ni-NTA) affinity chromatography (Qiagen). All buffers contained Complete EDTA-Free Protease Inhibitors (Roche) and manipulations were performed at 4°C. The resultant rPEG11 was assessed for purity and appropriate size using SDS-PAGE. Protein estimations were determined using the colorimetric bicinchoninic acid (BCA) kit with BSA as the standard (Pierce).

### Antibody production and purification

Antibodies were produced by four intramuscular injections of rabbits with rPEG11 (0.3 mg/dose) given subcutaneously over a 3 month schedule. The antibody specificity of the serum was validated by ELISA and immunoblotting using rPEG11 as the antigen. Antibody was immuno-affinity-purified according to a standard protocol using rPEG11 as the affinity protein [45] and then further purified by absorption through a glutathione S transferase affinity column.

### Nuclear preparations from skeletal muscle

Nuclear protein extracts were prepared from SM muscle samples taken from three *NN* and two *NC^pat^* animals at 12 weeks of age according to an established protocol [46] with minor modifications. Briefly, frozen SM muscle was wrapped in aluminium foil, snap frozen in liquid nitrogen and pulverised with a hammer. On ice, 4.0 g of the ground tissue was homogenized with 20 ml of 0.6% nonyl phenoxylpolyethoxylethanol (NP-40), 150 mM NaCl, 10 mM HEPES (pH 7.9), 1 mM EDTA, 0.5 mM PMSF and cellular debris pelleted by centrifugation (400×*g,* 30 s, 4^o^C). The supernatant containing intact nuclei was transferred to 40 ml centrifuge tubes, incubated on ice for 5 min, and centrifuged (10 min, 700×*g,* 4^o^C). The nuclei preparation was assessed by microscopy (400×magnification) using Trypan Blue staining. Nuclei stained as blue ovals visible either by themselves or attached to some remaining myofibril debris. The nuclear pellet was resuspended in 300 µl of 25% glycerol, 20 mM HEPES (pH 7.9), 420 mM NaCl, 1.2 mM MgCl_2_, 0.2 mM EDTA, 0.5 M DTT, 0.5 mM PMSF and 3 µl of a Complete Protease Inhibitor tablet (Roche) dissolved in 1 ml of H_2_O. The sample was placed on ice for 30 min and centrifuged (14000×*g*, 1 min, 4^o^C). The supernatant containing nuclear proteins was stored at −70°C. Protein quantification was performed using the NanoDrop ND-1000 spectrophotometer (Thermo Fischer Scientific).

### SDS-PAGE and Immunoblots

SDS-PAGE and immunobloting procedures were performed essentially according to previous descriptions [43]. Briefly, 100 µg of purified SM nuclear protein extract from each animal was added to sample buffer (60 mM Tris-HCl (pH 6.8), 2% SDS, 25% glycerol and 0.03% bromophenol blue, 5 mM DTT) and resolved on 6–18% gradient SDS polyacrylamide gels. The gels were then stained with a modified colloidal Coomassie blue G-250 stain [47]. For immunoblots, the separated proteins were transferred to Hybond-C nitrocellulose membrane (GE Healthcare Bio-Sciences) by electroblotting and then the membrane was blocked with TBS containing 0.1% Tween 20 and 1% (wt/vol) BSA for 30 min at room temperature. The membrane was incubated for 1 h with immunoaffinity-purified antibody to rPEG11 (0.1 µg/ml final concentration), followed by a second incubation with a biotinylated anti-rabbit IgG antibody (donkey anti-rabbit IgG biotinylated: GE Healthcare Bio-Sciences) (1∶7500, 1 h). After washing, a tertiary incubation was employed using an anti-biotin HRP linked antibody (Cell Signaling Technologies) (1∶2500 dilution, 1 h). SuperSignal West Pico Chemiluminescent Substrate (Pierce) was employed to visualise immunoreactive bands. Equal loadings on the gels were confirmed by colloidal Coomassie staining of a comparable gel.

### Tryptic digests

Following SDS-PAGE, protein regions corresponding to the immunoreactive bands of interest were excised from the gel, sliced into quarters and washed in distilled water. The gel pieces were washed (x3) with 50 µl of 100 mM ammonium bicarbonate and 50% acetoniltrile (ACN) (HPLC grade) in a sonicator for 20 min, followed by two washes with 50 µl of 40 mM ammonium bicarbonate. After solvent removal, the gel pieces were dried under vacuum at 30°C, rehydrated in 30 µl of digestion buffer (40 mM ammonium bicarbonate, 10% ACN) containing 600 ng (20 ng/µl) of sequencing grade modified trypsin (Promega) and incubated for 20 h at 37°C. The sample was centrifuged and the supernatant collected. The gel pieces were subsequently washed in 40 µl of 30% ACN and 5% formic acid followed by a second wash containing 60% ACN and 0.1% formic acid. All supernatants were combined and ACN removed under vacuum at 30°C. The peptides were reconstituted in 4 µl of 0.1% formic acid for LC-ESI-MS/MS analysis or 8 µl of 5% formic acid for LC-MALDI-TOF-TOF analysis. The peptide aliquots were sonicated for 20 min prior to analysis.

### LC-MALDI TOF-TOF

Tryptic peptides were fractionated using an Agilent 1100 series capillary HPLC System (Agilent), mixed with MALDI matrix (5 mg/ml of α–cyano-4-hydroxycinnamic acid in 50% ACN and 0.1% triofluoroacetic acid in milliQ water), and spotted directly onto an ABI 4700 OptiTOF sample plate. Briefly, peptide samples (∼8 µl) were injected onto a Vydac 300 Å C18 5 µm column (Vydac) pre-equilibrated with 0.1% formic acid (solvent A). Solvent B contained 90% ACN and 0.1% formic acid. HPLC separation of peptides was achieved using a linear gradient from 0 to 50% solvent B for 60 min followed by 50–80% solvent B for 10 min. Fractions were collected at 0.5 min intervals on a 192-position sample plate and simultaneously mixed with 0.5 µl of matrix. The samples were then subjected to MALDI MS/MS analysis using a 4700 Proteomics Analyzer equipped with TOF-TOF ion optics (Applied Biosystems) and 4000 Explorer version 3.6 analysis software. The instrument was operated in 1 kV positive ion reflector mode and calibrated with the 4700 Mass Standards kit (Applied Biosystems) for MS spectra and using Glu-fibrinopeptide B for MS/MS spectral calibration. The laser power was set to 5000 for MS and 5500 for MS/MS with CID off. MS spectra were acquired across the mass range of 850–4000 Da. MS/MS spectra were acquired manually for precursor ions with theoretical matches to the masses of tryptic peptides resulting from the PEG11 protein, with a total accumulation of 5000 laser shots.

### LC-ESI-MS/MS

ESI-MS data acquisition was performed using Analyst 1.4.1 software (Applied Biosystems) in the positive ion mode and multiple-reaction monitoring (MRM)-triggered acquisition of enhanced product ion (EPI) MS/MS spectra with enhanced resolution scan for charge state determination and precursor mass calculation. Chromatographic separation of tryptic peptides was achieved using an Ultimate 3000 HPLC system (Dionex) with a Phenomenex Luna C18, 5 µm (3.0 mm×250 mm) column. 10 µl of the tryptic digest was injected. A linear gradient from 0-40% B over 40 min was used. The eluent was coupled directly to a 4000 QTRAP (Applied Biosystems) mass spectrometer equipped with a TurboV source. An ion-spray voltage of 5,300 V was applied and N_2_ used as the curtain (value of 25) and collision gas (set to high), with the heated interface at 150°C. The de-clustering potential was set at 70 eV and Gas1 and Gas2 were each set to 35 psi. A 75 ms dwell time was used for each MRM transition. Following each survey scan, the top two precursor ions with multiple charge states with intensities greater than 150 counts/s were selected for MS/MS acquisition using a rolling collision energy based on the ions' observed charge states and masses.

### Mass spectrometry data analysis

To facilitate data interpretation, spectra from both mass spectrometers were searched against the Uniprot database (URL; downloaded in November 2007) that had been customized to include the putative PEG11 sequence with ProteinPilot version 2.0 (Applied Biosystems; Paragon search algorithm). The default search settings used for protein identification were trypsin cleavage with fixed iodoacetamide modification of cysteine. We report only protein identifications with a total ProtScore >1.3, which represents >95% statistical confidence in Protein Pilot. All peptide matches were manually verified.

### Analysis of PEG11 sequence

Sequence alignment was carried out using the ClustalW program [48]. The mammalian PEG11 coding sequences from the human, chimpanzee, orangutan, *Rhesus Macaca mulatta*, mouse, rat, guinea pig, cat, horse, cow and sheep genomes were extracted primarily by using the UCSC genome browser [34] as well as targeted searching of genomic sequence information not represented on this browser but present in the NCBI database (http://www.ncbi.nlm.nih.gov/). Conservation of the alignment of the nucleotide sequences at the 5′ ends of the longest open reading frames was used to identify the putative start codons. The ratio of nonsynonymous to synonymous substitutions, d_N_/d_S_, which is a measure of variable Darwinian selective pressures, was estimated by maximum likelihood from the PEG11 coding sequence alignment using the codeml program from PAMLv4 [49]. Ratios significantly greater than one indicate positive selection and ratios significantly less than one indicate purifying selection [50]. The relative positions of antisense miRNA in the *PEG11* nucleotide sequences and corresponding PEG11 protein sequences were deduced by using annotated miRNA identified in the UCSC browser, manual sequence inspection and literature information [31]. The ratio of non-synonymous change (d_N_) in these specific regions to the whole gene value of d_S_ was used to examine the influence of the antisense miRNA on the PEG11 coding sequence. Regions of *PEG11* sequence conservation in mammals were extracted using the UCSC PhastCons Conserved Elements Vertebrate Multiz Alignment and Conservation tool (http://genome.ucsc.edu/). Functional domains within the PEG11 protein sequence were identified using the Pfam Protein Families database (http://pfam.sanger.ac.uk/) [51]. Coiled coils were identified using Coils (http://www.ch.embnet.org/software/COILS_form.html) [52].
